# Performance of the Aptis Distal Radioulnar Joint Implant: A Clinical Case Series Including Four-Dimensional Computed Tomography Kinematic Analysis

**DOI:** 10.3390/jcm12185815

**Published:** 2023-09-07

**Authors:** Shirley D. Stougie, Margriet H. M. van Doesburg, Joris G. M. Oonk, Lara Plugge, Geert J. Streekstra, Johannes G. G. Dobbe, Jan Henk Coert

**Affiliations:** 1 Department of Plastic, Reconstructive and Hand Surgery, Amsterdam UMC, University of Amsterdam, Meibergdreef 9, 1105 AZ Amsterdam, The Netherlands; 2 Department of Plastic, Reconstructive and Hand Surgery, University Medical Center Utrecht, Heidelberglaan 100, 3584 CX Utrecht, The Netherlands; 3 Musculoskeletal Health—Restoration and Development, Amsterdam Movement Sciences, Meibergdreef 9, 1105 AZ Amsterdam, The Netherlands; 4 Department of Biomedical Engineering and Physics, Amsterdam UMC, University of Amsterdam, Meibergdreef 9, 1105 AZ Amsterdam, The Netherlands

**Keywords:** distal radioulnar joint, arthroplasty, functional outcomes, kinematics, complications

## Abstract

High complication rates and surgical revision rates following Aptis implant placement have been reported in the literature. This study evaluates the performance of the Aptis implant of twelve patients using four-dimensional kinematic analysis. The (mean) follow-up was 58 months. Wrist motion, grip strength, and kinematic analysis of both arms were used to investigate possible causes of the reported complications. In nine cases (75%), the proximal to distal translation of the distal radius along the ulnar axis in the affected forearm was too little or absent. Significant correlations were found between postoperative extension and translation of the distal radius along the ulnar axis and between the radial deviation and combined error. The four-dimensional kinematic analysis suggests that the current design of the implant could lead to limited restoration of the position of the forearm rotation axis and the translation of the radius along the ulnar axis.

## 1. Introduction

Wrist trauma resulting in a painful arthritic distal radioulnar joint (DRUJ) and instability can hinder activities of daily living. Many surgeons have performed resection or (partial) replacement of the ulnar head [1,2,3,4,5,6,7,8], which could lead to gross distal radioulnar instability and pain. To address these problems, Scheker [9] designed a (semi-) constrained implant to reconstruct the DRUJ to reduce pain and preserve functional wrist motion.

In the last decade, high complication rates and surgical revision rates (23–50%) have been reported [10,11,12,13,14,15,16,17,18,19]. Common complications reported are periprosthetic fracture, pisotriquetral arthritis [16], infection [17], periprosthetic extensor tendon, and extensor compartment problems [10,13,15]. Complications with periprosthetic extensor tendons and compartments may be explained by the malpositioning of the radial component of the implant in the distal region, which alters the kinematic axis of forearm rotation [20]. This may also be caused by the deformation of the distal radius.

Pääkkönen and Brannan [12,21] suggested that accurate surgical planning is required to guarantee correct placement of the implant. Data on wrist function, such as grip strength and wrist motion, may correlate with the position of the forearm rotation axis. Unfortunately, studies reporting these relations are scarce but may be valuable to find underlying causes for the reported complications.

Therefore, in this study, we evaluate the functional outcomes, the probable causes for the reported complications, and patient satisfaction following Aptis implant placement. We also correlate these functional parameters to parameters representing changes in forearm kinematics by using four-dimensional computed tomography and subsequent kinematic analysis.

## 2. Materials and Methods

### 2.1. Study Design

Patients who were treated with a unilateral Aptis implant between 2011 and 2021 were selected from our institutional database to participate in this present study. Patients with a minimum follow-up of six months and a healthy contralateral wrist were eligible for inclusion in the study. Exclusion criteria were a history of trauma and/or surgery of the healthy contralateral wrist. A letter of notification was sent to all eligible participants to announce the upcoming survey and were asked to take part in it. Subsequently, they were asked to provide their written informed consent. Included patients were subjected to comparisons of outcome measures between the affected wrist and healthy contralateral wrist.

Preoperative data extracted from electronic medical files in terms of active range of motion, pain score in the visual analog scale (VAS), and grip strength were not available in all patients to evaluate the result of the treatment. Postoperative data in terms of active range of motion and grip strength were collected and used for comparison between both arms and to establish possible correlations with kinematic data. This study was approved by the Medical Ethics Committee of our hospital.

### 2.2. Data Collection

After written informed consent was obtained, the patients completed the Dutch Language Version of the modified Patient Rated Wrist Hand Evaluation (PRWHE) questionnaire [22] and the Dutch–Flemish Language Version Patient Reported Outcome Measurement Information System (PROMIS) questionnaires [23]. The modified PRWHE questionnaire evaluates the pain, function, and aesthetics of the affected wrist. Higher scores indicate more pain and disability. The raw score for aesthetics is not a part of the scale scoring.

Additionally, the PROMIS questionnaires, which focus on ‘pain intensity’, ‘pain interference’, ‘upper extremity functioning’, ‘depression’, ‘anxiety’, and ‘ability to participate in social roles and activities’, evaluate global physical and mental health. A raw sum score per domain was converted with a PROMIS score service table into a T-score (www.healthmeasures.net/search-view-measures, accessed on 12 March 2023). The T-score is a standardized mean score of a large reference population. A higher PROMIS T-score correlates with a lower degree of similarity with the reference group. 

Electronic medical patient files were reviewed to extract the following data: gender, age, follow-up time, hand dominance, type of Aptis implant, affected wrist, relevant medical history of the contralateral healthy wrist, the number of previous surgeries, time between trauma and Aptis DRUJ arthroplasty, type of previous surgeries, indications, complications, revision surgery, X-rays and regular computed tomography (CT) scans, wrist range of motion of the affected wrist versus healthy contralateral wrist, grip strength of the affected wrist versus healthy contralateral wrist, pain score in the visual analog scale (VAS), and occupation. Patient satisfaction with the Aptis implant was determined using a 1 to 5 scale, indicating (1) very unsatisfied, (2) unsatisfied, (3) neutral, (4) satisfied, and (5) very satisfied. In addition, four (yes/no) questions were asked: Would you recommend the procedure to other patients? Would you choose the same procedure when in the same circumstances? Could you participate in your previous hobby? Could you return to your previous work? The complications following Aptis implant placement that are related to the implant were reported and graded by the Clavien–Dindo Classification [24].

### 2.3. Goniometric Measurement

**Figure 1:**
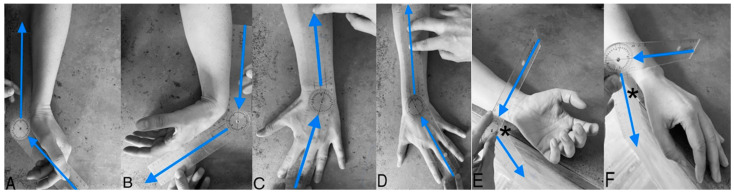
Goniometric measurement. (**A**) Extension; (**B**) Flexion; (**C**) Radial deviation; (**D**) Ulnar deviation; (**E**) Supination; (**F**) Pronation. (*) The edge of the table was used to find the line perpendicular to the table surface, which serves as reference in measuring pronation and supination. Note: the arrows indicate the fulcrum of the wrist goniometer to find the angle of wrist motion.

Patients were examined by a blinded experienced hand therapist in the outpatient clinic to collect postoperative data on the active range of motion and grip strength of the affected wrist and the healthy contralateral wrist. Wrist motion was measured with a universal manual wrist goniometer (Figure 1). The patient was seated and asked to position their elbow in 90 degrees flexion and the forearm in the neutral position stabilized on the table. 

### 2.4. Grip Strength Measurement

**Figure 2:**
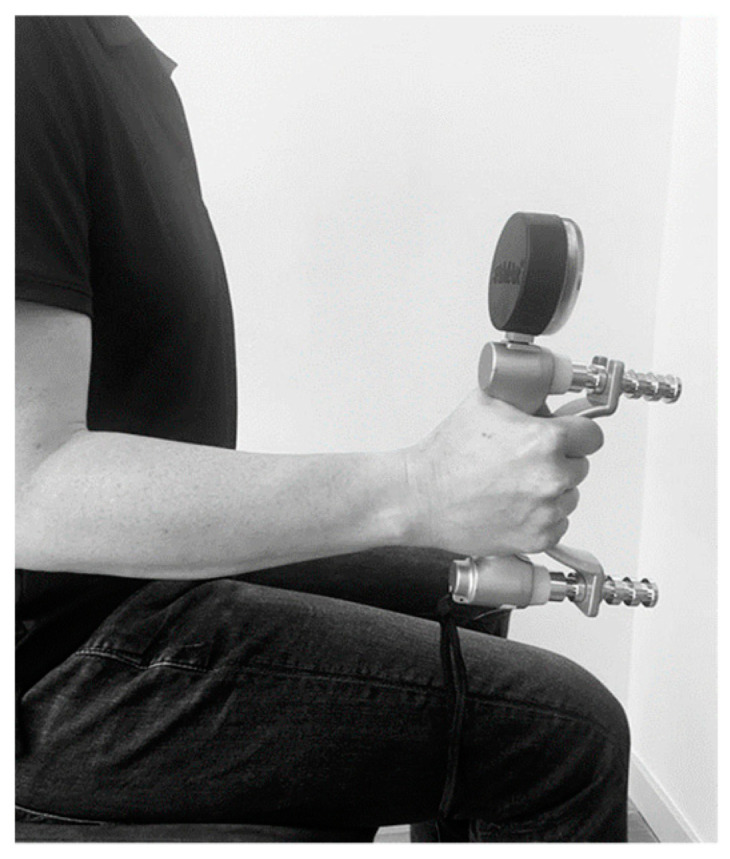
Grip strength measurement.

Grip strength was measured using a Jamar hand dynamometer in handle position two (Figure 2). The patients were still seated with their elbow in 90 degrees flexion and the forearm in the neutral position without stabilizing their forearm on the table. The patient was asked to exert as much force as they could, and a total of six measurements were taken, alternating between left and right until each side had completed three measurements. The average of the three measurements was the final grip strength value for that side.

### 2.5. Radiographic Assessment

Postoperative X-rays were made at follow-up to evaluate bone and implant positioning, signs of loosening or migration, heterotopic ossifications, periprosthetic radio-lucencies, or fractures. Oonk [20] investigated the gross performance of the Aptis implant for the same cohort as in the present paper. A selection of his results was used to investigate possible correlations between his kinematic findings and the functional parameters in the present study. For kinematic analysis, an additional computed tomography (CT) scan and four-dimensional CT (4DCT) scans were made to compare the motion of the treated wrist with the contralateral healthy wrist. These 4DCT scans were made of the DRUJ (or midshaft, see Oonk et al. [20]) and proximal distal radioulnar joint (PRUJ) region for the arm with Aptis implant in pronation–supination motion and of the wrist in flexion–extension motion. This was repeated for the contralateral healthy arm, which served as a kinematic reference. The bones were then segmented from the high-quality CT scan, and (parts of) these bones are registered to the subsequent time frames of each 4DCT scan. This provided: (1) motion data of each segmented bone, (2) a ‘combined error’ parameter representing malpositioning of the forearm rotation axis, represented by a displacement of the rotation axis at the DRUJ level, and (3) translation of the distal radius during pronation–supination motion, as measured along the central axis of the ulna.

### 2.6. Surgical Technique and Postoperative Treatment

Twelve second-generation Aptis implants (Aptis Medical, Louisville, KY, USA) were placed by one senior surgeon with an experience level V, as reported by Nakamura [25]. The procedural steps of surgery were performed in the standard fashion, as described in detail by Scheker [9]. However, a small modification of the original surgical technique was performed because the DRUJ was approached with a straight incision through the fifth extensor compartment, and the implant was covered by closing the fifth extensor compartment. In three cases, an ulnar head implant (Herbert, KLS Martin Medizin, Tuttlingen, Germany) was removed, and in one case, a hemi-ulnar head implant (Ascension, TX, USA) was removed.

Postoperative treatment consisted of seven days of short arm casting and self-rehabilitation. The patients were allowed to start with active mobilization of the wrist in all directions. Gradual weight bearing was allowed, with a maximum weight of up to fifteen kilograms three months postoperatively.

### 2.7. Statistics

Descriptive analyses were used to describe the study cohort in terms of patient satisfaction, complication rates, and surgical revision rates. Differences in pre- and postoperative active range of motion and grip strength between the affected wrist and the contralateral healthy wrist, and pain scores in the VAS at rest and during activities, were compared using the paired Wilcoxon signed rank test for data that were not normally distributed. The Shapiro–Wilk test was used to test for normality as well for the PRWE scores, PROMIS scores, position of the forearm axis, and the difference in the translation of the radius along the ulnar axis in the affected wrist compared to that of the healthy contralateral wrist. Spearman’s correlations were calculated on ordinal data to study associations between satisfaction, pain, complications, wrist motion, the position of the forearm rotation axis, the difference in the translation of the radius along the ulnar axis, and PRWE scales pain, function, and total. Spearman’s rank test was used to calculate correlations between patient satisfaction, complications, PRWE scores, and PROMIS T-scores. *p* values < 0.05 were considered statistically significant.

## 3. Results

### 3.1. Demographics and Follow-Up

Twelve patients participated in the study to compare their forearms with an Aptis implant with the healthy contralateral forearm, including a four-dimensional computed tomography (CT) evaluation for kinematic analysis of their forearms. The demographic characteristics and surgical characteristics in this cohort were similar (Table 1). This cohort consisted of eight men and four women with a mean age of 50 years at surgery (range: 26–65 years). The dominant hand was operated on in six patients. All twelve patients had undergone surgery before the Aptis distal radioulnar joint (DRUJ) arthroplasty (range: 1–5 and mean of 2.9 surgeries in the wrist). The mean time between wrist trauma and Aptis DRUJ arthroplasty was 70.1 months (range: 13–231 months). The main indication was post-traumatic DRUJ osteoarthritis and ulnar instability (*n* = 4), ulnar instability after an (hemi-) ulnar head implant (*n* = 4), ulnar instability after a Sauvé–Kapandji procedure, and primary DRUJ osteoarthritis (*n* = 3). Patients were evaluated in January 2023 at a (mean) follow-up time of 58 months (range: 13–113 months).

### 3.2. Clinical Evaluation

**Figure 3:**
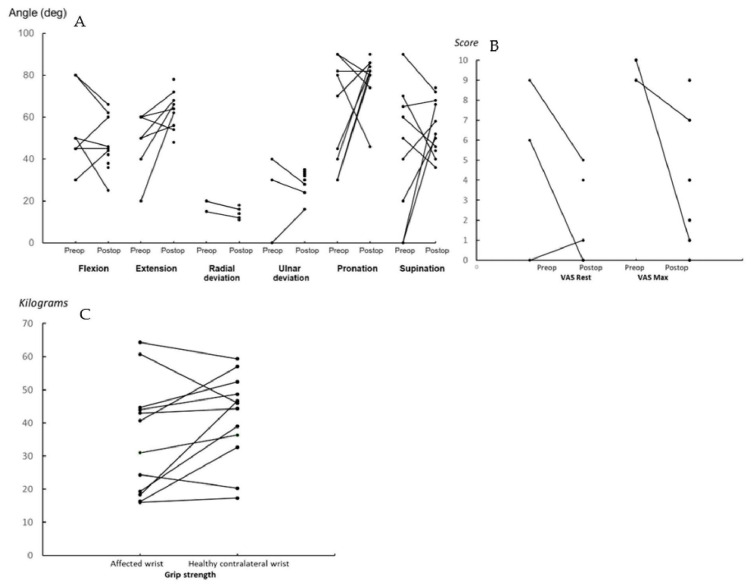
(**A**) Preoperative and postoperative motion in degrees of the affected wrist. (**B**) Follow-up VAS pain score at rest and during activities (maximum). (**C**) Follow-up grip strength affected wrist and contralateral healthy wrist.

An overview of the clinical results of all patients is presented in Table 2. The differences in preoperative and postoperative wrist motion and pain score in the VAS are presented in Figure 3A,B. The differences in postoperative grip strength of the affected wrist and the healthy contralateral wrist are presented in Figure 3C.

A minimal decrease in the radial deviation was observed (*p* = 0.008) in three cases. 

The mean pain score in the visual analog scale (VAS) at rest and during activity improved considerably in about half of the patients. 

The median grip strength was 35.8 kg in the affected wrist and 45.2 kg in the healthy contralateral wrist.

Patient satisfaction with the implant is presented in Table 3, and patient-rated outcome measurements are presented in Table 4. The mean postoperative Patient-Rated Wrist Hand Evaluation scores indicate insufficient benefits from pain and functioning since the norm is 7.7 in healthy subjects [26]. 

The mean PROMIS T-scores indicate good global physical and mental health in all cases.

### 3.3. Radiological Evaluation

An overview of the X-ray examination and the kinematic analysis is presented in Table 5. It has been shown that in nine cases (75%), the proximal to distal translation of the distal radius along the ulnar axis in the affected forearm was too little or absent. Only in two cases (5 and 11) was the translation roughly about the same as in the healthy contralateral forearm.

**Figure 4:**
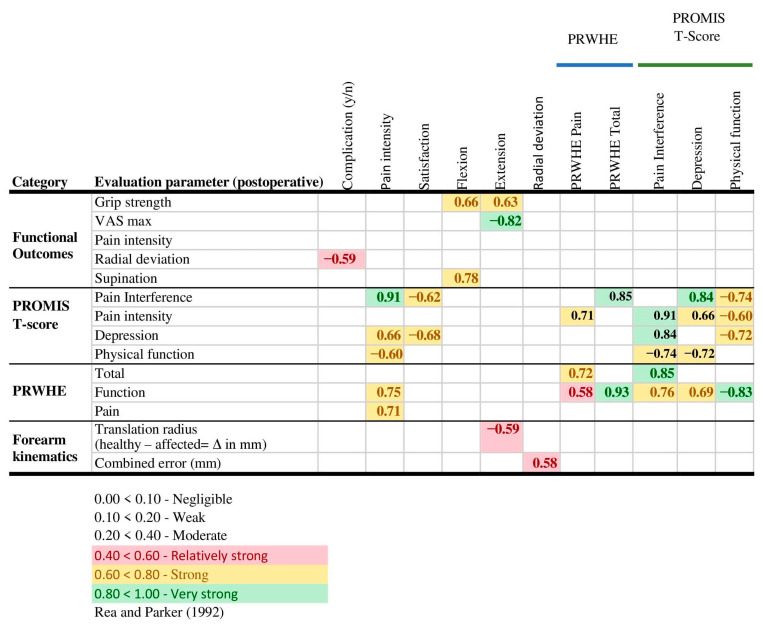
Evaluation of correlations according to Rea and Parker [27] postoperative parameters. Abbreviations: Δ, difference.

Significant Spearman’s correlations are presented in Figure 4. As expected, a significant negative correlation was found between patient satisfaction and PROMIS T-score ‘pain interference’ and ‘depression’. A significant positive correlation was found between PROMIS T-score ‘depression’ and pain intensity, PRWHE Function, PRWHE total, and PROMIS T-scores ‘pain interference’ and ‘physical function’. And, as expected, a significant positive correlation was found between pain intensity and PRWHE pain, PRWHE Function, PRWHE total, and the PROMIS T-scores ‘depression’, ‘pain interference’, and ‘physical function’.

Interestingly, significant positive correlations were found between postoperative radial deviation and complications. Moreover, significant correlations were found with kinematic parameters, namely, between postoperative extension and translation of the distal radius along the ulnar axis, and between radial deviation and the combined error, indicating an effect of a malpositioned forearm rotation axis. As expected, a significant negative correlation was found between postoperative extension and postoperative pain.

### 3.4. Complications and Revision Surgery

Complications and surgical revisions are presented in Table 6. The kinematic findings resulting from 4DCT analysis for cases 1, 2, 3, and 7 are described below in more detail to explain possible causes for complications requiring revision surgery.

**Figure 5:**
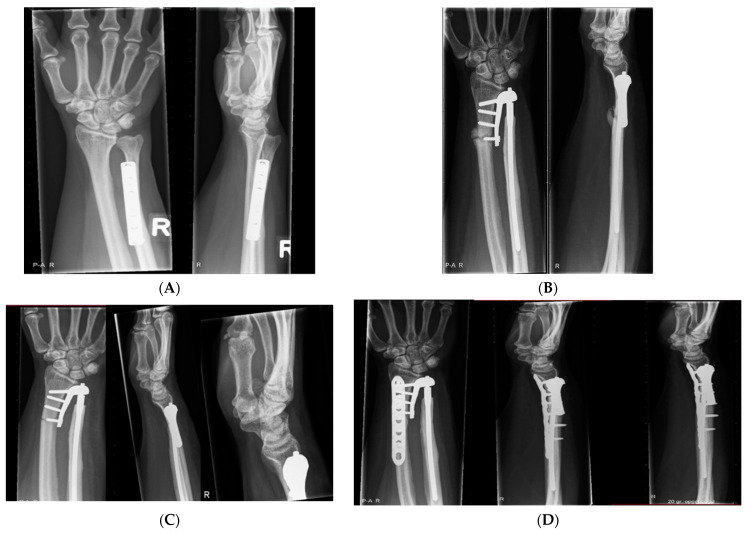

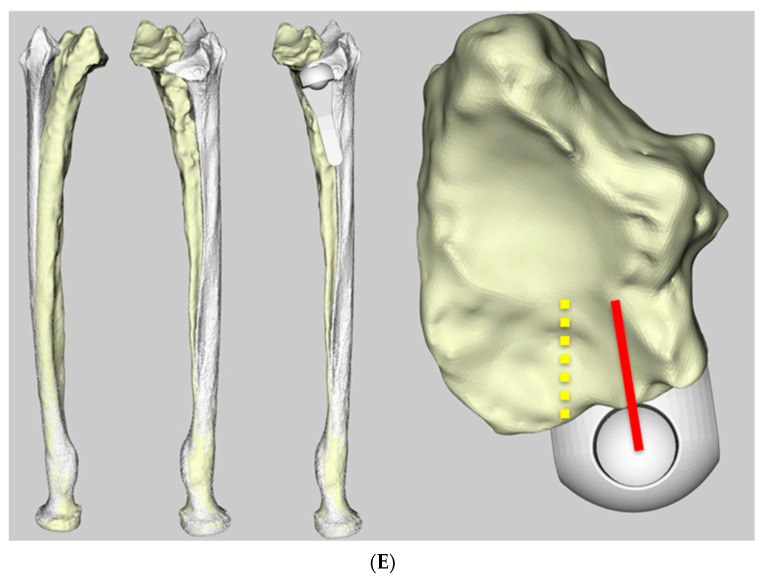
(**A**) Lateral and anteroposterior X-rays of case 1 before implantation. (**B**) Lateral and anteroposterior X-rays of case 1 18 days after implantation. (**C**) Lateral and anteroposterior X-rays of case 1. (**D**) Lateral and anteroposterior X-rays of case 19 months after implantation. (**E**) Three-dimensional CT reconstruction of the radius and the position of the implant. Note, the curvature of the radius and the dorsal placement of the implant (red). Yellow illustrates the purely ulnar ward position of the implant according to the Aptis manual.

A 54-year-old woman (case 1) with a history of ulnar shortening osteotomy (Figure 5A), denervation of the DRUJ, and an Aptis implant presented with severe wrist pain due to a periprosthetic distal radius fracture (Figure 5B) 18 days postoperatively, most likely as a result of poor bone stock. Unfortunately, there was a non-union of the distal radius (Figure 5C) after 10 weeks of short arm casting. The patient was finally treated with a volar locking plate fixation (Figure 5D), and the clinical consequences were pain and limited rotation. 

In this case, the radial component of the implant was placed dorsally on the ulnar side of the radius due to the length and curvature of the radius (Figure 5E). This implant position could have resulted in a combined error of 11 mm and a decreased translation of the radius along the ulnar axis (−1.0 mm) compared to the healthy contralateral forearm.

**Figure 6:**
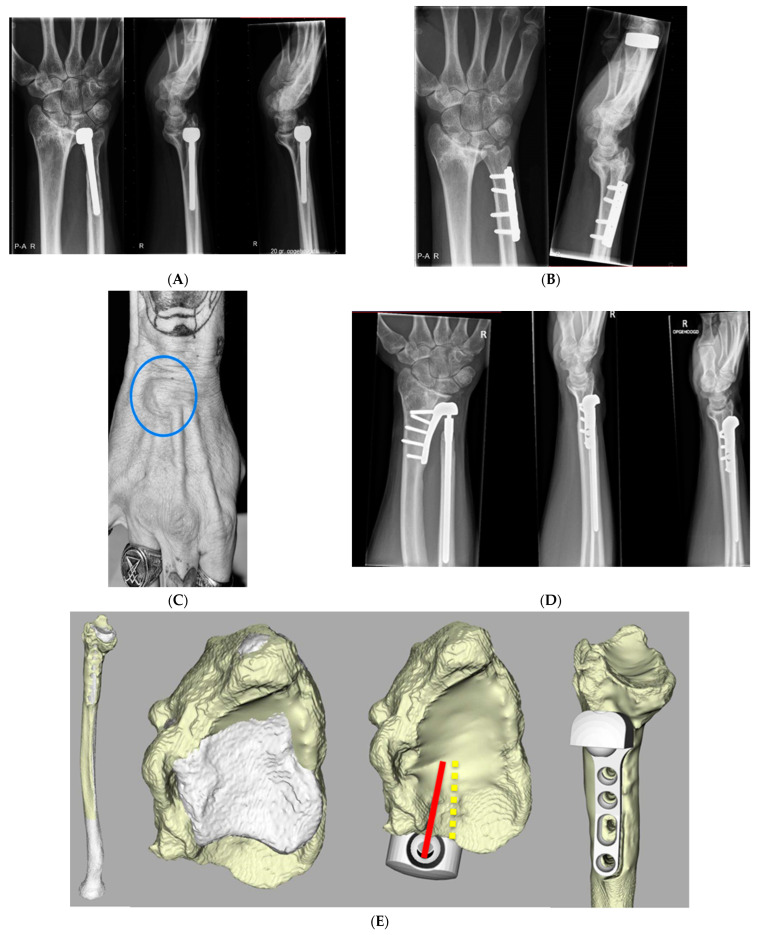
(**A**) Lateral and anteroposterior X-rays of case 2 before hemi-ulnar head implantation. (**B**) Lateral and anteroposterior X-rays of case 2 before Aptis DRUJ implantation. (**C**) Clinical example of tenosynovitis of the fourth extensor compartment of the right wrist (blue circle). (**D**) Lateral and anteroposterior X-rays of case 2 9.5 years after Aptis DRUJ implantation. (**E**) Three-dimensional CT reconstruction of radius and the position of the implant (Case 2). Note, the remodeling of the radius and the dorsal placement and dorsal rotation of the implant (red). Yellow illustrates the purely ulnar ward position of the implant according to the Aptis manual.

A 43-year-old man (case 2) with a history of a forearm fracture, ulnar shortening osteotomy (Figure 6A), (hemi-) ulnar head implant (Figure 6B), and an Aptis implant complicated with an extensor carpi ulnaris (ECU) tendinitis presented 9.5 years after the implantation with severe wrist pain and swelling at the dorsum of the right wrist (Figure 6C). X-rays (Figure 6D) and an ultrasound examination were conducted and tenosynovitis of the fourth extensor compartment was observed, most likely as a result of attrition. A tenosynovectomy was performed, and during this procedure it was observed that attrition of the extensor tendons had occurred for approximately 50%.

The implant was placed in a relatively dorsal position on the ulnar side of the radius with a dorsal rotation towards the radius (Figure 6E), probably dictated by the volar ridge of the sigmoid notch as a result of a malunited radius. Most likely the surgeon had to choose a relatively dorsal placement on the ulnar side of the radius to allow for a proper fit of the radial component and its screws to avoid friction forces which may cause breaking out of the screws at the radial cortical surface. 

**Figure 7:**
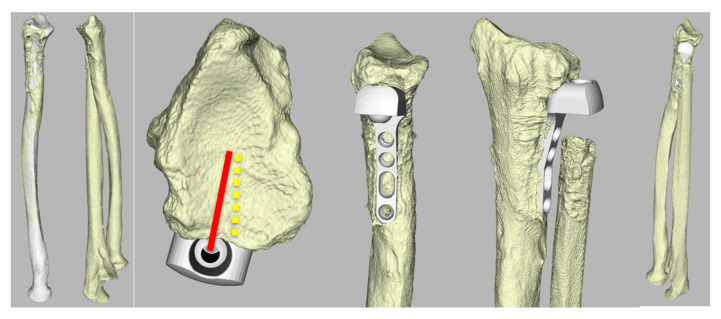
Three-dimensional CT reconstruction of radius and the position of the implant (case 3). Note, the remodeling of the radius and the dorsal placement and dorsal rotation of the implant (red). Yellow illustrates the purely ulnar ward position of the implant according to the Aptis manual.

A 47-year-old man (case 3) with a history of left-sided Galeazzi fracture, corrective osteotomy of the radius, capsulodesis of the DRUJ, tightrope stabilization procedure of the DRUJ, and finally an Aptis implant presented with severe radial wrist pain 3.5 months postoperatively as a result of a protruding screw and/or irritation of a small metal plate from the tightrope. In one single session, a first extensor compartment release with the removal of the metal plate and shortening of the screw was performed successfully. Unfortunately, seven months after the implantation an ECU tendinitis occurred, which was treated adequately with rest and short arm casting. Eighteen months after the implantation, a posterior interosseous nerve neurectomy was performed due to persisting wrist pain during pronation. At the 45-month follow-up, the patient still experienced considerable wrist pain despite a reasonable forearm motion. X-rays showed radiolucency around the peg, most likely as a result of a low-grade infection, which was treated with antibiotics. The implant was placed relatively dorsally and rotated towards the radius (Figure 7), which could have resulted in a decreased translation of the radius along the ulnar axis (−1.1 mm) compared to the healthy contralateral forearm.

A 65-year-old man (case 7) with a history of wrist trauma, a Sauvé–Kapandji procedure, an ulnar head implant, and finally an Aptis implant presented with severe wrist pain and limited rotation five months after the implantation. Hereby, the distal ulna fusion was left in situ. The Aptis DRUJ arthroplasty was complicated by ECU tendinitis and extreme heterotopic ossifications around the implant, which were treated adequately with the removal of the heterotopic ossifications.

**Figure 8:**
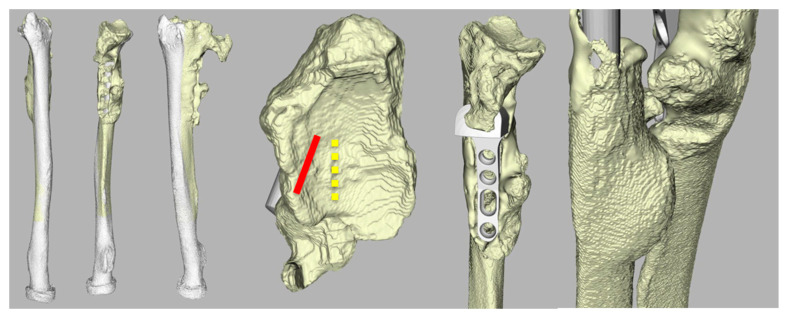
Three-dimensional CT reconstruction of radius and the position of the implant (case 7). Note, the remodeling of the radius, the dorsal placement and dorsal rotation (red) of the implant, and the extreme heterotopic ossifications just proximal of the implant. Yellow illustrates the purely ulnar ward position of the implant according to the Aptis manual.

The position of the implant was relatively dorsal on the ulnar side of the radius with a dorsal rotation towards the radius (Figure 8) which could have resulted in a combined error of 14 mm and a decreased translation along the ulnar axis (−1.9 mm) compared to the healthy contralateral forearm. 

## 4. Discussion

The study evaluates the performance of the Aptis implant in a clinical case series including twelve patients using four-dimensional kinematic analysis. Four-dimensional CT analysis of both forearms was performed to assess the position of the implant, the forearm rotation axis, and translation of the radius along the ulnar axis during pronation–supination motion. The kinematic parameters of the affected forearm were compared with the healthy contralateral forearm to investigate possible causes for the reported complications. 

Wrist motion and grip strength were compared between the affected wrist and the healthy contralateral wrist and correlated with the position of the forearm rotation axis and the difference in the translation of the radius along the ulnar axis. The results have shown improvement in wrist motion, less pain, good grip strength, good patient satisfaction, good Patient Rated Wrist Evaluation, and a good PROMIS evaluation, yet a high complication rate and surgical revision rate have also been shown. The follow-up revealed multiple periprosthetic, extensor tendons, and compartment problems, as well as surgical revisions in cases with altered forearm kinematics. Interestingly, translation of the distal radius along the centerline of the ulna decreased in nine cases (75%). In five cases with extensor carpi ulnaris tendinitis were heterotopic ossifications seen on the X-rays. In all cases with heterotopic ossifications, the combined error was increased considerably compared to the cases without abnormalities seen on the X-rays. A significant negative correlation was found between postoperative extension and postoperative pain. Wrist extension increased in six cases. Considerable pain during activities remained in six cases. Nevertheless, most patients would undergo the procedure again or recommend it to others. This is comparable with the findings by Rampazzo [28] and Lambrecht [29].

The high incidence of extensor tendon and compartment problems is similar to the findings of Stougie [10], Warlop [13], DeGeorge [15], and Rampazzo [28]. 

Surgical revisions were required to address the problems encountered with extensor tendons and compartments after relatively dorsal placement in the distal region of the radius and a dorsal rotation of the distal implant towards the radius. 

Noteworthy, heterotopic ossifications and extensor carpi ulnaris tendinitis (ECU) were seen in all cases with the conversion from an ulnar head implant to an Aptis implant. This could suggest that instability after an ulnar head implant could result in heterotopic ossifications with an ECU as a clinical consequence.

The incidence of surgical revision should be avoidable by secure screw placement and the use of a preoperative custom-designed drilling and saw guide to allow optimal surgical placement of the implant. Based on the CT scan, a 3D model of the affected radius can be generated and used for the virtual placement of the screws and the design of the drilling guide. Multiple cases with complications showed a relatively dorsal placement and a dorsal rotation of the implant most likely as a result of radius deformity. In cases with severe radius deformity, it could be a viable option to perform a corrective osteotomy of the radius first to reduce the risk of malpositioning of the radial component in the distal region.

In this study, preoperative examination parameters, such as active range of motion, grip strength, and expectations and indications for surgery, were not available for all participants, which can be considered a limitation. As a result, patient satisfaction, PRWE scores, and PROMIS scores were asked retrospectively, which may cause recall bias among the respondents. The small cohort is heterogeneous, and this may affect the clinical results, especially in patients with multiple surgeries or after ulnar head implant revision, since the possible effect of multiple surgeries on the soft tissue (i.e., higher risk of heterotopic ossifications or dystrophic calcifications). The statistical analysis was limited to reporting descriptive statistical parameters. Finally, we had a large variation in the follow-up duration. For future studies, it would be of great value to investigate why the implant is more likely to fail in certain patients. 

In conclusion, a periprosthetic fracture, extensor tendon, and compartment problems occurred in the cases with heterotopic ossifications and in which volar and purely ulnar ward placement of the implant was not possible due to radius deformity or remodeling of the radius. Wrist extension increased in these cases, whilst flexion decreased. The four-dimensional kinematic analysis suggests that the current design of the implant could lead to the limited restoration of the position of the forearm rotation axis and the translation of the radius along the ulnar axis. Aptis DRUJ arthroplasty is a viable treatment option, but revision surgery might be needed in cases with an extensive operative history.

## Figures and Tables

**Table 1:** Demographics characteristics, follow-up in months, and surgical characteristics.

Case	Age (Years)	Gender M/F	Follow-Up Time	Dominant Hand	Affected Hand	Time between Trauma and Implantation	Previous Surgeries	Indication
1	54	F	37	R	R	-	3	DRUJ OA
2 *	35	M	113	R	R	81	4	Ulnar instability after (hemi-) UH implant
3	43	M	45	R	L	60	3	DRUJ OA and instability
4	63	F	76	R	R	29	1	DRUJ OA and instability
5 *	55	M	59	R	R	-	4	Ulnar instability after UH implant
6	59	F	13	R	L	13	3	DRUJ OA
7 *	65	M	40	R	L	231	5	Ulnar instability after UH implant
8	56	M	20	R	L	14	3	DRUJ OA and instability
9	26	M	44	R	L	90	3	Ulnar instability after SK procedure
10	62	M	47	R	R	96	1	DRUJ OA
11	38	M	106	L	L	17	2	DRUJ OA and instability
12 *	56	F	95	R	L	-	5	Ulnar instability after UH implant
Mean (SD)	50.4 (13.3)		57.9 (32.7)			70.1 (69.0)	3.1 (1.3)	

Abbreviations: DRUJ, distal radioulnar joint; OA, osteoarthritis; * ulnar head implant (U-head) removed; UH, ulnar head; SK, Sauvé–Kapandji.

**Table 2:** Evaluation clinical results (preoperative/postoperative).

Category	Wrist Motion Affected Side						Wrist Motion Healthy Contralateral Side						Grip Strength Pain		
Case	Flexion °	Extension °	Radial Deviation °	Ulnar deviation °	Supination °	Pronation °	Flexion °	Extension °	Radial Deviation °	Ulnar Deviation °	Supination °	Pronation °	R/L (kg) Postoperative	VAS Pain Rest	VAS Pain Max
1	45/46	60/54	-/16	-/32	60/46	80/46	66	64	20	36	64	90	16.0/17.3	-/4	-/9
2	80/62	40/66	-/14	-/28	20/50	90/80	80	80	30	38	74	86	64.3/59.3	-/0	-/4
3	50/25	50/56	20/16	30/24	50/36	70/86	60	72	22	40	40	80	46.7/18.3	-/4	-/7
4	-/42	-/72	-/12	-/30	-/50	-/82	62	68	28	38	50	80	24.3/20.3	-/0	-/0
5	-/62	-/64	-/14	-/35	0/66	30/82	60	65	22	36	60	110	60.7/46.0	6/0	10/1
6	50/46	60/64	-/16	-/34	40/58	45/84	56	68	23	38	66	84	36.3/31	0/1	-/2
7	80/66	60/72	20/16	40/28	90/72	90/74	76	74	18	30	62	98	48.7/44	-/0	-/0
8	45/60	50/68	-/11	-/33	65/68	70/86	90	80	18	32	62	85	52.5/44.7	-/1	-/4
9	-/46	-/64	-/18	-/30	-/74	-/90	62	70	26	12	76	90	39/19.3	9/5	9/7
10	-/36	-/78	-/14	-/28	-/44	-/84	59	78	16	30	52	82	43/44.3	-/0	-/0
11	30/44	20/62	15/12	0/16	0/52	30/80	60	80	18	30	60	90	57/40.7	10/1	10/4
12	-/38	-/48	-/16	-/34	70/40	40/86	68	33	17	33	64	84	32.7/16.3	-/0	-/1
Mean (SD)	54.2 (18.8)/ 49.7 (14.2)	43.3 (14.6)/ 63.3 (6.4)	18.3 (2.9)/ 14.7(2.3)	23.3 (20.8)/ 22.7 (6.1)	43.9 (31.6)/ 54.2 (12.7)	62.7 (24.1)/ 78.4 (12.0)	66.9 (10.3)	69.3 (12.9)	21.5 (4.5)	32.8 (7.4)	60.8 (9.9)	88.3 (8.6)		6.3 (4.5)/ 1.8 (2.2)	9.7 (0.6)/ 4.0 (3.0)
*p*-value	0.464	0.051	0.08 ‡	0.945	0.381	0.135								0.122	0.108

Abbreviations: °, degrees; R, right; L, left; kg, kilograms; max, maximum; ‡, statistically significant.

**Table 3:** Patient satisfaction, occupation, return to hobby/work.

Case	Patient Satisfaction with Aptis Implant	Aptis Implant Again?	Recommend Aptis Implant	Occupation	Return Work/Hobby
1	Neutral	No	No	Not able to work	No/No
2	Very satisfied	Yes	Yes	Navy	Yes/Yes
3	Very satisfied	Yes	Yes	Volunteer	No/No
4	Very satisfied	Yes	Yes	Retired	NA/Yes
5	Very satisfied	Yes	Yes	Manual Laborer	Yes/Yes
6	Very satisfied	Yes	Yes	Photographer	Yes/Yes
7	Very satisfied	Yes	Yes	Retired	NA/Yes
8	Very satisfied	Yes	Yes	Lieutenant Fire Fighter	Yes/Yes
9	Very satisfied	Yes	Yes	Courier	No/Yes
10	Very satisfied	Yes	Yes	Retired	NA/Yes
11	Satisfied	Yes	Yes	Account Manager	Yes/Yes
12	Neutral	Yes	Yes	Domestic help	Yes/Yes
%	(83%)	(92%)	(92%)		(83%)/(50%)

Abbreviations: NA, not applicable.

**Table 4:** Follow-up PRWE scores and PROMIS scores after Aptis DRUJ arthroplasty.

PRWHE Scores					PROMIS T-Scores (SE)					
Case	Pain	Function	Total	Esthetics	Pain Intensity Raw-Score	Pain Interference	Physical Function Upper Extremity	Depression	Anxiety	Ability to Participate in Social Roles + Activities
1	40	33.5	77.5	4 *	8	71 (1.4)	35.6 (2.4)	62.1 (1.8)	63.5 (2.0)	47.0 (1.6)
2	9	3	12	0	1	40.7 (5.9)	47.7 (3.9)	38.2 (5.7)	43.2 (3.3)	65.4 (4.9)
3	20	44	64	0	5	64.1 (1.3)	24.9 (2.3)	50.9 (2.0)	49.4 (2.3)	36.9 (1.5)
4	0	0	0	0	0	40.7 (5.9)	58.2 (6.7)	38.2 (5.7)	37.1 (5.5)	65.4 (4.9)
5	4	0	4	0	1	51.2 (1.5)	58.2 (6.7)	38.2 (5.7)	37.1 (5.5)	52.7 (1.6)
6	8	10	18	0	1	52.3 (1.4)	38.6 (2.6)	44.7 (3.3)	47.8 (2.5)	56.8 (1.7)
7	1	1.5	2.5	0	0	40.7 (5.9)	58.2 (6.7)	44.7 (3.3)	47.8 (2.5)	58.2 (2.0)
8	12	3	15	0	2	49.9 (1.8)	50.9 (4.5)	38.2 (5.7)	37.1 (5.5)	51.7 (1.6)
9	33	20.5	53.5	0	5	57.4 (1.3)	45.6 (3.6)	44.7 (3.3)	47.8 (2.5)	52.7 (1.6)
10	0	8.5	8.5	0	0	40.7 (5.9)	40.9 (2.9)	38.2 (5.7)	37.1 (5.5)	65.4 (4.9)
11	14	11.5	25.5	0	2	54.1 (1.4)	45.6 (3.6)	44.7 (3.3)	43.2 (3.3)	56.8 (1.7)
12	4	7	13	0	2	58.8 (1.3)	34.7 (2.4)	56.8 (1.7)	56.4 (2.0)	40.2 (1.6)
Mean (SD)	9.1 (9.7)	11.9 (14.0)	25.6 (27.9)		2.25 (2.49)			44.97 (7.97)		
Median (IQR)						51.75 (40.7–58.8)	45.6 (36.4–56.4)		45.5 (37.1–49)	54.8 (48.2–63.6)

Abbreviations: PRWE, Patient Rated Wrist Evaluation (indicating 0 points for no pain and being able to do activities, 100 points for worst pain imaginable and not being able to do activities); * unsatisfied with the appearance of the hand/wrist; PROMIS, Patient Rated Outcome Measure Information System (T-score of 50 points is the average for the general population in the United States with a standard deviation of 10); SE, standard error; SD, standard deviation; IQR, interquartile range.

**Table 5:** Radiological follow-up (months), differences in the forearm rotation axes (mm) at the level of the DRUJ, and differences in translation of the radius along the ulnar axis (mm).

Case	Radiological Evaluation	Combined Error	Translation Healthy	Translation Aptis	Δ Translation
1	Periprosthetic fracture; proximal screws	11	1.5	0.5	−1.0
2	Lucency distal screw (37)	6	2.8	0.6	−2.2
3	Heterotopic ossification (8)	7	1.3	0.2	−1.1
4	No abnormalities	5	2.8	0.5	−2.3
5	Heterotopic ossification (4)	12	2.7	3.5	0.8
6	-	7	1.4	4.1	2.7
7	Heterotopic ossification (5) + Lucency distal screw radius (13)	14	2.1	0.3	−1.9
8	No abnormalities	6	1.6	0.3	−1.3
9	Chip distal ulna (7) + Heterotopic ossification (24)	13	1.8	0.3	−1.5
10	Dystrophic calcification (1.5)	10	2.4	0.4	−2.0
11	Lucency distal screw (8) + heterotopic ossification (48)	10	1.9	2.0	0.1
12	Heterotopic ossification (57)	9	1.7	0.3	−1.4
Average (SD)		9 (3)	2.0 (0.6)	1.1 (1.3)	−0.9 (1.4)

Abbreviations: SD, standard deviation; Δ, difference.

**Table 6:** Complications identification after Aptis DRUJ arthroplasty, revision surgery, Clavien–Dindo classification.

Case	Complications (Months)	Revision Surgery	Clavien–Dindo Classification
1	Periprosthetic fracture; proximal screws (18 days postoperative)	Volare locking plate fixation Removal osteosynthesis material Posterior interosseus nerve neurectomy	Grade IIIa Grade IIIa Grade IIIa
2	Recurrent ECU tendinitis (37) Tenosynovitis fourth extensor compartment (113)	None Tenosynovectomy	Grade IIIa
3	Protruding screw (3.5) ECU tendinitis (8) Painful rotation (18)	Shortening screw tip and release first extensor compartment Posterior interosseus nerve neurectomy	Grade IIIa Grade IIIa
4	None	None	
5	ECU tendinitis (4)	None	Grade II
6	None	None	
7	Extreme heterotopic ossification	Removal heterotopic ossification	Grade IIIa
8	None	None	
9	ECU tendinitis (7)	None	Grade II
10	Adhesion FPL after removal volar locking plate radius	None	
11	None	None	
12	ECU tendinitis (13) + (57)	None	Grade II

## Data Availability

Not applicable.

## References

[1] Kachooei A.R., Chase S.M., Jupiter J.B. (2014). Outcome assessment after Aptis Distal Radioulnar Joint (DRUJ) implant arthroplasty. Arch. Bone Jt. Surg..

[2] Willis A.A., Berger R.A., Cooney W.P. (2007). Arthroplasty of the distal radioulnar joint using a new ulnar head endoprosthesis: Preliminary report. J. Hand Surg. Am..

[3] Van Schoonhoven J., Kall S., Schober F., Prommersberger K.J., Lanz U. (2003). The hemiresection-interposition arthroplasty as a salvage procedure for the arthritically destroyed distal radioulnar joint. Handchir. Mikrochir. Plast. Chir..

[4] Sauerbier M., Hahn M.E., Fujita M., Neale P.G., Berglund L.J., Berger R.A. (2002). Analysis of dynamic distal radioulnar convergence after ulnar head resection and endoprosthesis implantation. J. Hand Surg. Am..

[5] Darrach W. (1992). Partial excision of lower shaft of ulna for deformity following Colles’s fracture. Clin. Orthop. Relat. Res..

[6] Watson H.K., Ryu J.Y., Burgess R.C. (1986). Matched distal ulnar resection. J. Hand Surg. Am..

[7] Bell M.J., Hill R.J., McMurtry R.Y. (1985). Ulnar impingement syndrome. J. Bone Jt. Surg. Br..

[8] Swanson A.B. (1973). Implant arthroplasty for disabilities of the distal radioulnar joint. Use of a silicone rubber capping implant following resection of the ulnar head. Orthop. Clin. N. Am..

[9] Scheker L.R., Babb B.A., Killion P.E. (2001). Distal ulnar prosthetic replacement. Orthop. Clin. N. Am..

[10] Stougie S.D., Van Boekel L., Beumer A., Hoogvliet P., Strackee S.D., Coert J.H. (2023). Aptis distal radioulnar joint arthroplasty: A multicenter evaluation of functional outcome, complications and patient satisfaction. J. Wrist Surg..

[11] Amundsen A., Rizzo M., Berger R.A., Houdek M.T., Frihagen F., Moran S.L. (2023). Twenty-year experience with primary distal radioulnar joint arthroplasty from a single institution. J. Hand Surg. Am..

[12] Brannan P.S., Ward W.A., Gaston R.G., Chadderdon R.C., Woodside J.C., Connell B. (2022). Two-year clinical and radiographic evaluation of Scheker prosthesis (Aptis) distal radioulnar joint arthroplasty. J. Hand Surg. Am..

[13] Warlop J., Van Nuffel M., De Smet L., Degreef I. (2021). Midterm functional outcomes of the linked semiconstrained distal radioulnar joint prosthesis. J. Wrist Surg..

[14] Fuchs N., Meier L.A., Giesen T., Calcagni M., Reissner L. (2020). Long-term results after semiconstrained distal radioulnar joint arthroplasty: A focus on complications. Hand Surg. Rehabil..

[15] DeGeorge B.R., Berger R.A., Shin A.Y. (2019). Constrained implant arthroplasty for distal radioulnar joint arthrosis: Evaluation and management of soft tissue complications. J. Hand Surg. Am..

[16] Lans J., Chen S.H., Jupiter J.B., Scheker L.R. (2019). Distal radioulnar joint replacement in the scarred wrist. J. Wrist Surg..

[17] Bellevue K.D., Thayer M.K., Pouliot M. (2018). Complications of semiconstrained distal radioulnar Joint arthroplasty. J. Hand Surg. Am..

[18] Moulton L., Giddins G. (2017). Distal radio-ulnar implant arthroplasty: A systematic review. J. Hand Surg. Eur..

[19] Calcagni M., Giesen T.F. (2016). Distal radioulnar joint arthroplasty with implants: A systematic review. EFORT Open Rev..

[20] Oonk J.G.M., Dobbe J.G.G., Strijkers G.J., Van Rijn S.K., Streekstra G.J. (2023). Kinematic analysis of forearm rotation using four-dimensional computed tomography. J. Hand Surg. Eur..

[21] Pääkkönen M. (2022). Complications of Scheker semiconstrained distal radioulnar joint arthroplasty in a low-volume unit. Hand Surg. Rehabil..

[22] MacDermid J.C., Tottenham V. (2004). Responsiveness of the disability of the arm, shoulder, and hand (DASH) and patient-rated wrist/hand evaluation (PRWHE) in evaluating change after hand therapy. J. Hand Ther..

[23] Hays R.D., Bjorner J.B., Revicki D.A., Spritzer K.L., Cella D. (2009). Development of physical and mental health summary scores from the patient-reported outcomes measurement information system (PROMIS) global items. Qual. Life Res..

[24] Clavien P.A., Barkun J., de Oliveira M.L., Vauthey J.N., Dindo D., Schulick R.D., de Santibañes E., Pekolj J., Slankamenac K., Bassi C. (2009). The Clavien-Dindo classification of surgical complications: Five-year experience. Ann. Surg..

[25] Nakamura T. (2020). Surgeons’ Level of Expertise. J. Wrist Surg..

[26] Mulders A.M., Kleipool S.C., Dingemans S.A., Van Eerten P., Schepers T., Goslings J.C., Schep N.W.L. (2018). Normative data for the Patient-Rated Wrist Evaluation questionnaire. J. Hand Ther..

[27] Rea L.M., Parker R.A. (1992). Designing and Conducting Survey Research: A Comprehensive Guide.

[28] Rampazzo A., Gharb B.B., Brock G., Scheker L.R. (2015). Functional outcomes of the Aptis-Scheker Distal Radioulnar Joint Replacement in Patients Under 40 years old. J. Hand Surg. Am..

[29] Lambrecht D., Vanhove W., Hollevoet N. (2022). Clinical and radiological results of distal radioulnar joint arthroplasty with the Aptis prosthesis. J. Hand Surg. Eur..

